# Propofol Ameliorates H9c2 Cells Apoptosis Induced by Oxygen Glucose Deprivation and Reperfusion Injury via Inhibiting High Levels of Mitochondrial Fusion and Fission

**DOI:** 10.3389/fphar.2019.00061

**Published:** 2019-02-12

**Authors:** Lidong Zhao, Jinqiang Zhuang, Yihui Wang, Dandan Zhou, Dandan Zhao, Shun Zhu, Jinjun Pu, Hongyu Zhang, Ming Yin, Wenjuan Zhao, Zejian Wang, Jiang Hong

**Affiliations:** ^1^Department of Internal and Emergency Medicine, Shanghai General Hospital, Shanghai Jiao Tong University School of Medicine (Originally Named “Shanghai First People’ s Hospital”), Shanghai, China; ^2^Department of Emergency Medicine, Putuo Hospital Affiliated to Shanghai University of Traditional Chinese Medicine, Shanghai, China; ^3^Department of Biomedicine, KG Jebsen Centre for Research on Neuropsychiatric Disorders, University of Bergen, Bergen, Norway; ^4^School of Pharmacy, Shanghai Jiao Tong University, Shanghai, China

**Keywords:** propofol, ischemia, reperfusion, mitochondrial dynamics, apoptosis

## Abstract

**Background:** The cardioprotective effect of propofol on ischemia-reperfusion injury (I/R injury) is partly due to suppressing apoptosis. Mitochondrial dynamics are also involved in apoptosis. Mitochondrial fusion and fission lead to mitochondrial morphological changes. However, whether suppressing apoptosis effect of propofol against ischemia-reperfusion injury in the heart is via regulating mitochondrial morphology remains unclear.

**Methods:** H9c2 cells underwent oxygen glucose deprivation (OGD) followed by reperfusion to simulate cardiomyocytes ischemia/reperfusion injury. Cell viability, apoptosis ratio and intracellular reactive oxygen species (ROS) were assessed, respectively. Mitochondrial membrane dynamin family proteins, extracellular signal regulated kinase 1 and 2 (ERK1/2), phosphorylated extracellular signal regulated kinase 1 and 2 (p-ERK1/2) and proteins related to intrinsic apoptosis pathways were detected by western blotting. The mitochondrial morphology and the distribution of dynamin-related protein 1 (Drp1) were observed by using laser confocal microscopy.

**Results:** Propofol enhanced the survival of H9c2 cells, decreased ROS levels and inhibited apoptosis during oxygen glucose deprivation/reperfusion (OGD/R) injury. Mitochondrial fission in H9c2 cells was inhibited by propofol during OGD injury. Propofol alleviated high levels of mitochondrial fusion and fission during OGD/R in H9c2 cells, by regulating mitochondrial membrane remodeling dynamin family proteins. Propofol inhibited Drp1 colocalization with mitochondria in H9c2 cells during OGD/R injury. Moreover, Drp1 phosphorylation was inhibited by propofol through decreasing ERK activation during OGD/R injury. We found that propofol ameliorated H9c2 cells apoptosis during OGD/R via inhibiting mitochondrial cytochrome c release and caspase-9, caspase-6, caspase-7 and caspase-3 activation.

**Conclusion:** Propofol suppresses H9c2 cells apoptosis during OGD/R injury via inhibiting intrinsic apoptosis pathway, which may be partly due to reducing high levels of mitochondrial fusion and fission induced by OGD/R injury.

## Introduction

The intravenous anesthetic propofol (2, 6-diisopropylphenol) is widely used in anesthesia and sedation due to the rapid induction and recovery from anesthesia (Wahr et al., 2000). Many lines of evidence indicate that propofol exhibits cardioprotective effects (Kokita and Hara, 1996; Xia et al., 2003). As for the mechanism, similar to vitamin E, propofol contains a phenolic hydroxyl group, which has been associated with its antioxidant effect (Vasileiou et al., 2009). Researches indicate that propofol can scavenge the free radical, both *in vitro* and *in vivo* (Li et al., 2012), thus ameliorating ischemic myocardial contractile dysfunction and arrhythmias (Hanouz et al., 2003), narrowing infarct size, and reducing tissue lesions (Ko et al., 1997). Moreover, propofol has been shown to attenuate ischemia-reperfusion injury (I/R injury) by suppressing apoptosis and preserving mitochondrial function (Jin et al., 2009), but the exact mechanism remains unclear.

Mitochondria are the most important sources of energy in the heart, providing over 90% adenosine triphosphate (ATP) to the heart through oxidative phosphorylation (Schaper et al., 1985). In addition, mitochondria also play a key role in regulating apoptosis and cell growth, and in generating reactive oxygen species (ROS). Additionally, mitochondrial morphology is now recognized as an important factor closely associated with the energetic state of mitochondria (Galloway et al., 2012b). Mitochondrial morphology varies among different cell types. Mitochondria are in the process of continuous fission and fusion mediated by membrane remodeling dynamin family proteins (Ishihara et al., 2009). When oxidative stress occurs during acute I/R injury, mitochondrial fission can be caused in HL-1 cardiac cells (Ong et al., 2010). Dynamin family proteins involve mitofusin 1 (Mfn1), mitofusin 2 (Mfn2) and optic atrophy 1 (Opa1) protein that mediate mitochondrial fusion, whereas dynamin-related protein (Drp1) and fission 1 (Fis1) protein regulate mitochondrial fission.

Cardiomyocyte apoptosis plays an essential role in acute myocardial ischemia-reperfusion injury (I/R injury) (Haunstetter and Izumo, 1998). Apoptosis can be regulated through both intrinsic and extrinsic pathways (Zhang et al., 2002). Mitochondrial-shaping proteins are involved in intrinsic apoptosis pathway (Ong et al., 2017). They play important roles in the mitochondrial outer membrane permeabilization (MOMP) and the release of apoptotic factors, for example, cytochrome c release (Montessuit et al., 2010). However, whether suppressing apoptosis effect of propofol against ischemia-reperfusion injury (I/R injury) in the heart is via an intrinsic mitochondrial mechanism remains unclear. Based on previous studies, we hypothesize that propofol may reduce cardiomyocyte apoptosis induced by acute ischemia-reperfusion injury (I/R injury), via an intrinsic mitochondrial mechanism, by regulating mitochondrial fusion and fission. In this study, we used the H9c2 cell line subjected to oxygen glucose deprivation (OGD) followed by reperfusion (OGD/R) as an *in vitro* model of cardiomyocytes ischemia and investigated the underlying mechanism of propofol against cells apoptosis.

## Materials and Methods

### Cell Culture and Reagents

The H9c2 cells, a cardiomyocyte cell line, were purchased from the Shanghai Institute for Biological Sciences, Chinese Academy of Sciences (Shanghai, China). Dulbecco’s modified Eagle’s medium/F-12 (DMEM/F-12) and fetal bovine serum (FBS) were both purchased from Gibco-Invitrogen (Grand Island, NY, United States). The cells were cultured in DMEM/F-12, supplemented with 10% FBS and 1% penicillin/streptomycin at 37°C in a humidified incubator containing 95% air and 5% CO_2_.

### Oxygen Glucose Deprivation (OGD)/Reoxygenation (OGD/R) Model and Drug Treatment

H9c2 cells were incubated with a normal medium in a cell incubator for 24 h. Cells were then exposed to hypoxic conditions (oxygen deprivation, 1% O_2_) for 24 h in a culture medium with lower glucose and 1% FBS. After hypoxia, the cells were oxygenated under a normal oxygen concentration (reoxygenation) for 24 h in a normal medium. According to the previous study, propofol (Sigma-Aldrich, United States) was added respectively to the cells 1 h before and during the hypoxia-reoxygenation with different concentrations (5, 10, and 20 μM) (Zhao et al., 2015). U0126 (Beyotime, Haimen, China), MEK1/2 inhibitor, was added to the cells during OGD/R at a concentration of 20 μM. After H9c2 cells were incubated with a normal medium in cell incubator for 24 h, propofol and caspase inhibitor Z-VAD-FMK (Beyotime, Haimen, China) were respectively added to the cells for 24 h.

### Cell Viability Assay

Cell viability was measured by a cell counting kit-8 (CCK-8), purchased from Tokyo, Japan. Cells were seeded in 96-well cell culture plates at a density of 2 × 10^4^ cells/well. After 24 h in the culture, cells were added with different concentrations of propofol for hypoxia-oxygenation or just were respectively added with different concentrations of propofol and Z-VAD-FMK. Then, the supernatant fluid was removed and a medium containing 10% CCK- 8 reagent was added for 1–3 h incubation at 37°C. Eventually, the absorbance at 450 nm was measured using an AMR-100 automatic enzyme analyzer (Allsheng, Hangzhou, China).

### Intracellular ROS Detection

In order to detect intracellular ROS, the cells were preloaded with 10 μM 2, 7-dichlorofluorescin diacetate (DCFH-DA, Beyotime, Haimen, China) for 20 min at 37°C. Afterward, the cells were washed using a culture medium without serum for at least three. A fluorescence microplate reader with an excitation wavelength of 488 nm and an emission wavelength of 525 nm was used to determine the intensity of DCF fluorescence.

### Cell Apoptosis Assay

Cells were seeded into a 6-well cell culture plate and an established OGDR model, and treated with propofol. The FITC Annexin V Apoptosis Detection Kit (BD Pharmingen^TM^, United States) was used to detect apoptotic cells according to the manufacturer’s protocol. First, cells were washed twice with cold PBS and then cells were resuspended in a 1X Binding Buffer at a concentration of 1 × 10ˆ6 cells/ml. Second, 100 μl of the solution (1 × 10ˆ5 cells) was transferred to a 5 ml culture tube. Third, 5 μl of FITC Annexin V and 5 μl PI was added. Fourth, the cell were gently vortexed and incubated for 15 min at room temperature (25°C) in the dark. Fifth, 400 μl of the 1X Binding Buffer was added to each tube. Lastly, the cells were analyzed via flow cytometry within 1 h. The proportion of apoptotic cells was calculated by FlowJo software.

### Confocal Laser Scanning Fluorescence Microscopy

Confocal fluorescence microscopy was performed using a Laser Scanning Confocal Microscope (TCS SP5; Leica Microsystems, Inc., United States). After cells climbed to the carry sheet glass, we established an OGDR model and treated cells with propofol. The cells were then incubated for 15 min at room temperature with a 100 nM Mito-Tracker Red (Invitrogen, United States) to stain the mitochondrial membrane. Cells were fixed in 4% paraformaldehyde for 15 min and blocked with 5% bovine serum albumin (BSA) for 2 h, and then permeabilized with Triton-X 100 (0.1%) for 15 min. Cells were incubated with the primary mouse anti-Drp1 antibody (1:100) (BD Biosciences, United States) at 4°C overnight, washed with cold PBS, and incubated with Fluorescein-conjugated anti-mouse secondary antibody (1:100) (Proteintech, Wuhan, China) for 2 h at room temperature. The cells were then imaged at x630 magnification. Image J software was used to analyze the mitochondrial fragmentation count.

### Mitochondria/Cytosol Fractionation

This assay was conducted using a Mitochondrial Isolation Kit (Pierce, Rockford, IL, United States) according to the manufacturers’ protocol. Cells were homogenized and the homogenates were centrifuged at 750 *g* for 10 min at 4°C. The pellet was kept as a mitochondrial fraction. The supernatant was centrifuged at 12,000 *g* for 15 min at 4°C. The supernatant was further centrifuged at 100,000 *g* for 1 h, and the supernatant was kept as the cytosol fraction. Cytochrome c in cytoplasm or mitochondria was detected with an immunoblot analysis, respectively.

### Western Blotting

Cell lysates were separated on 6, 10, 12% SDS-PAGE, and transferred to polyvinylidene difluoride membranes (PVDF, Millipore, Billerica, MA, United States). The membranes were blocked and incubated with primary antibodies against anti-Drp1 (1:1000, BD Biosciences, United States), anti-pDrp1 (1:1000, CST, United States), anti-Fis1 (1:1000, Proteintech, China), anti-Mfn1 (1:1000, Proteintech, China), anti-Mfn2 (1:1000, CST, United States), anti-Opa1 (1:1000, BD Biosciences, United States), anti-ERK (1:1000, CST, United States), anti-pERK (1:1000, CST, United States), anti-Cytochrome c (1:1000, CST, United States), anti-Bax (1:1000, CST, United States), anti-Bak (1:1000, CST, United States), anti-Apaf1 (1:1000, Proteintech, China), anti-Caspase 9 (1:1000, CST, United States), anti-Caspase 6 (1:1000, CST, United States), anti-Caspase 7 (1:1000, CST, United States), anti-Caspase3 (1:1000, CST, United States), anti-COX IV (1:1000, CST, United States) and anti-GAPDH (1:1000, CST, United States) at 4°C overnight. After primary antibody incubation, the membranes were incubated with either goat anti-mouse or goat anti-rabbit horseradish peroxidase-conjugated secondary antibodies, at room temperature for 2 h. Immunoreactive bands were developed with an electrochemiluminescence (ECL) reagent (Thermo-Pierce, Rockford, IL, United States). Images were quantified using Gel-Pro Analyzer software (Media Cybernetics, Silver Spring, MD, United States).

### Statistical Analysis

The results were presented as the mean ± standard deviation (SD). Statistical analysis was performed using the software Statistical Package for the Social Science (SPSS, Chicago, IL, United States). The analysis of variance (ANOVA) was used for comparisons among groups and differences with a *P* < 0.05, were considered statistically significant.

## Results

### Propofol Enhanced the Survival of H9c2 Cells and Decreased the ROS Level Against OGD/R Injury. Moreover, the H9c2 Cells Proliferation Under Normal Culture Conditions Was Inhibited by Propofol and Caspase Inhibitor Relieved the Effect of Propofol

**FIGURE 1:**
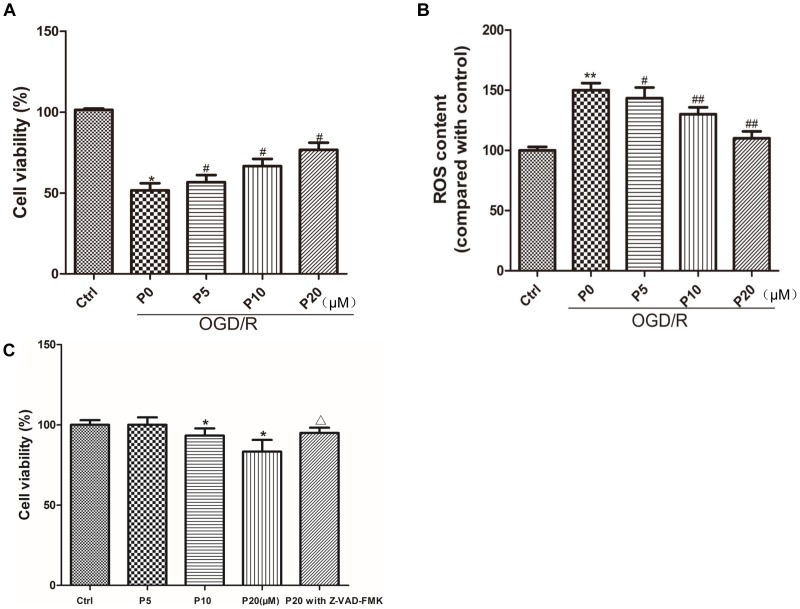
Propofol enhanced the survival of H9c2 cells and decreased ROS level against OGD/R injury. Moreover, H9c2 cells proliferation under normal culture conditions was inhibited by propofol and caspase inhibitor relieved the effect of propofol. **(A,C)** Cell viability was assessed by measuring the CCK8. The viability of control group was defined as 100%. **(B)** Effects of propofol (5, 10, and 20 μM) on increased intracellular ROS induced by OGD/R in H9c2 cells. The ROS contents of control group was defined as 100. The results were shown as mean ± SD from at least three independent experiments. ^∗^*P* < 0.05, ^∗∗^*P* < 0.01 versus control, ^#^*P* < 0.05, ^##^*P* < 0.01 versus OGD/R group without propofol, ^Δ^*P* < 0.05 versus 20 μM propofol group.

Compared with the control group, cell viability was significantly decreased in the OGD/R group without propofol. But as the concentration of propofol (5, 10, and 20 μM) went up, H9c2 cells survival increased compared with the OGD/R group without propofol (Figure 1A). In this experiment, OGD/R obviously elevated the intracellular ROS level compared with control group. Interestingly, compared with the OGD/R group without propofol, the ROS level was obviously inhibited with the increasing dose of propofol (5, 10, and 20 μM) (Figure 1B). These results demonstrated that propofol inhibited cell death and decreased the intracellular ROS level induced by OGD/R in the H9c2 cells. Moreover, propofol (10 and 20 μM) inhibited the H9c2 cells proliferation under normal culture conditions and Z-VAD-FMK could relieve the decrease of cell viability induced by 20 μM propofol (Figure 1C). These results demonstrated that high concentrations of propofol could cause damage to the H9c2 cells by activating caspases.

### Propofol Inhibited Cell Apoptosis Induced by OGD/R in H9c2 Cells

**FIGURE 2:**
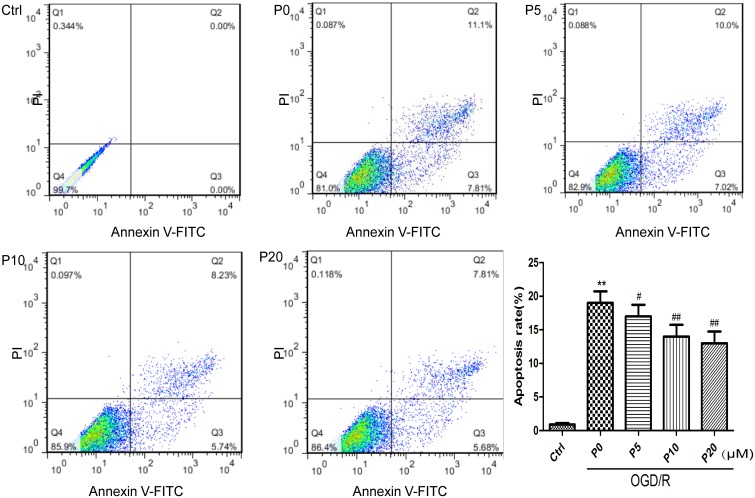
Propofol significantly inhibited H9c2 cells apoptosis induced by OGD/R injury. The cells were analyzed by flow cytometry. The data are presented as the mean ± SD of at least three independent experiments. ^∗∗^*P* < 0.01 versus control, ^#^*P* < 0.05, ^##^*P* < 0.01 versus OGD/R group without propofol.

Cardiomyocyte apoptosis in acute myocardial ischemia-reperfusion injury could be one of the main mechanisms. The results of the present study demonstrated that H9c2 cell apoptosis in the OGD/R group without propofol, was significantly increased compared with the control group. Compared with the OGD/R group without propofol, propofol alleviated H9c2 cell apoptosis induced by OGD/R, in a dose-dependent manner, at the range of 5∼20 μM (Figure 2). These results were consistent with a previous report that propofol could attenuate cardiomyocytes apoptosis induced by H_2_O_2_ (Liu et al., 2017).

### Propofol Inhibited Mitochondrial Fission in H9c2 Cells During OGD Injury

**FIGURE 3:**
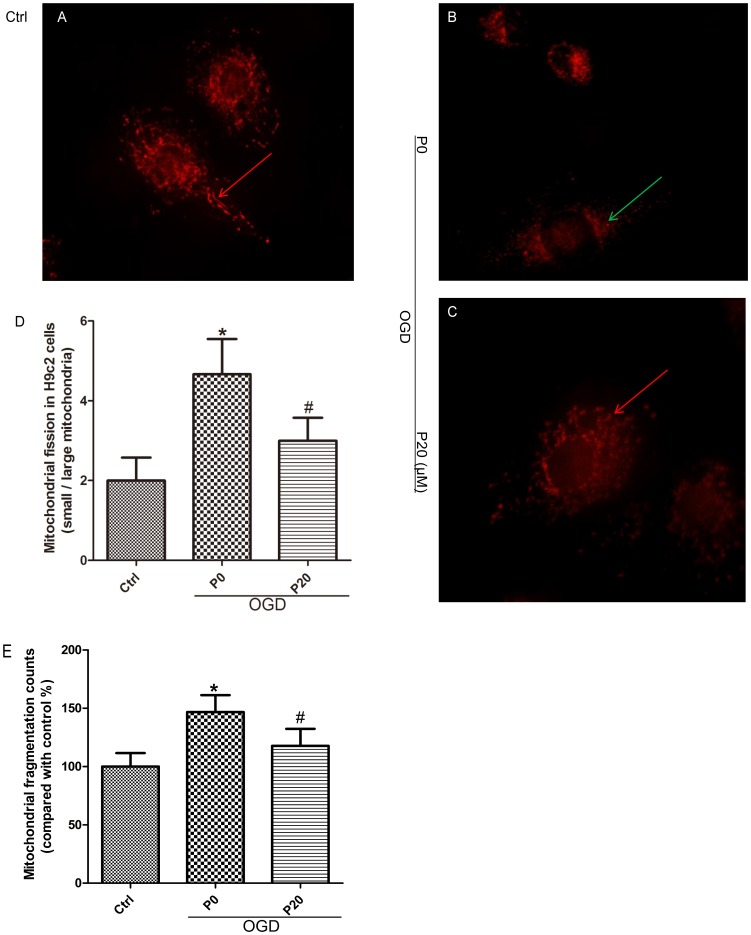
Propofol inhibited mitochondrial fission in H9c2 cells during OGD injury. **(A,B,D,E)** Compared with control group, small mitochondria (green arrows) increased in H9c2 cells during OGD injury. Scale bar: 10 μm. **(B–E)** Propofol increased the quantity and proportion of large mitochondria (red arrows) in H9c2 cells during OGD injury. The data are presented as the mean ± SD of at least three independent experiments. ^∗^*P* < 0.05 versus control, ^#^*P* < 0.05, versus OGD group without propofol.

When oxidative stress occurs during acute I/R injury, mitochondrial fission can be caused in HL-1 cardiac cells (Ong et al., 2010). Compared with the control group, mitochondrial fragmentation was obvious, and the quantity of mitochondria increased in H9c2 cells during OGD injury (Figure 3A,B,D,E). Compared with the OGD group without propofol, propofol (20 μM) significantly inhibited mitochondrial fission and reduced the quantity of mitochondria (Figure 3B–E).

### Propofol Alleviated High Levels of Mitochondrial Fusion and Fission During OGD/R in H9c2 Cells by Regulating Mitochondrial Membrane Remodeling Dynamin Family Proteins

**FIGURE 4:**
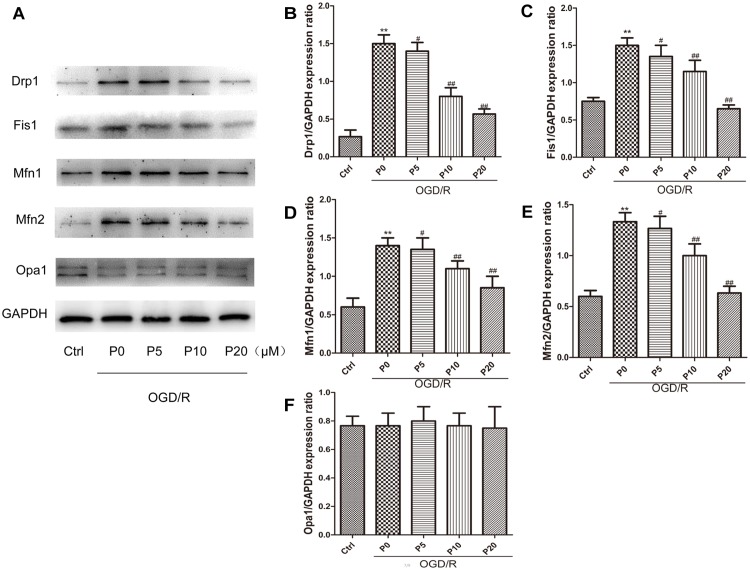
Propofol inhibited high expression of some mitochondrial dynamin family proteins induced by OGD/R in H9c2 cells. **(A)** Representative bands of Drp1, Fis1, Mfn1, Mfn2 and Opa1 by western blotting. **(B–E)** With the increase of propofol concentration (5, 10, 20 μM), the levels of Drp1, Fis1, Mfn1 and Mfn2 protein were significantly decreased during OGD/R injury. **(F)** However, there was no significant difference in the levels of Opa1 between control group and OGD/R group with or without propofol. The data are presented as the mean ± SD of at least three independent experiments. ^∗∗^*P* < 0.01 versus control, ^#^*P* < 0.05, ^##^*P* < 0.01 versus OGD/R group without propofol.

It is well-accepted that Drp1 and Fis1 proteins promote mitochondrial fission, whereas Mfn1, Mfn2 and Opa1 proteins mediate mitochondrial fusion. Furthermore, high expression of Drp1, Mfn1 and Mfn2 may lead to cell death in the heart (Whelan et al., 2012; Park et al., 2014). The experimental results demonstrated that in the OGD/R group without propofol, the levels of Drp1 and Fis1 proteins were significantly elevated compared with the control group. Moreover, with the increase of propofol concentration, the levels of Drp1 and Fis1 proteins were significantly decreased during OGD/R injury (Figure 4A–C). These results may suggest that propofol could inhibit the high expression of mitochondrial fission proteins induced by OGD/R injury. Compared with the control group, the levels of Mfn1 and Mfn2 proteins were also significantly elevated during OGD/R injury. Additionally, with the increase of propofol concentration, the high levels of Mfn1 and Mfn2 proteins during OGD/R injury were significantly decreased (Figure 4A,D,E). However, there was no significant difference in the levels of Opa1 between the control and OGD/R without propofol group. Propofol did not influence the levels of Opa1 during OGD/R injury (Figure 4A,F). Propofol may also inhibit the high levels of some mitochondrial fusion proteins induced by OGD/R injury. The experimental results demonstrated that mitochondrial fission and mitochondrial fusion were significantly elevated by OGD/R injury and were alleviated by propofol.

### Mitochondrial Morphology and Quantity Kept Stable and Propofol Inhibited Drp1 Colocalization With Mitochondria in H9c2 Cells During OGD/R Injury

**FIGURE 5:**
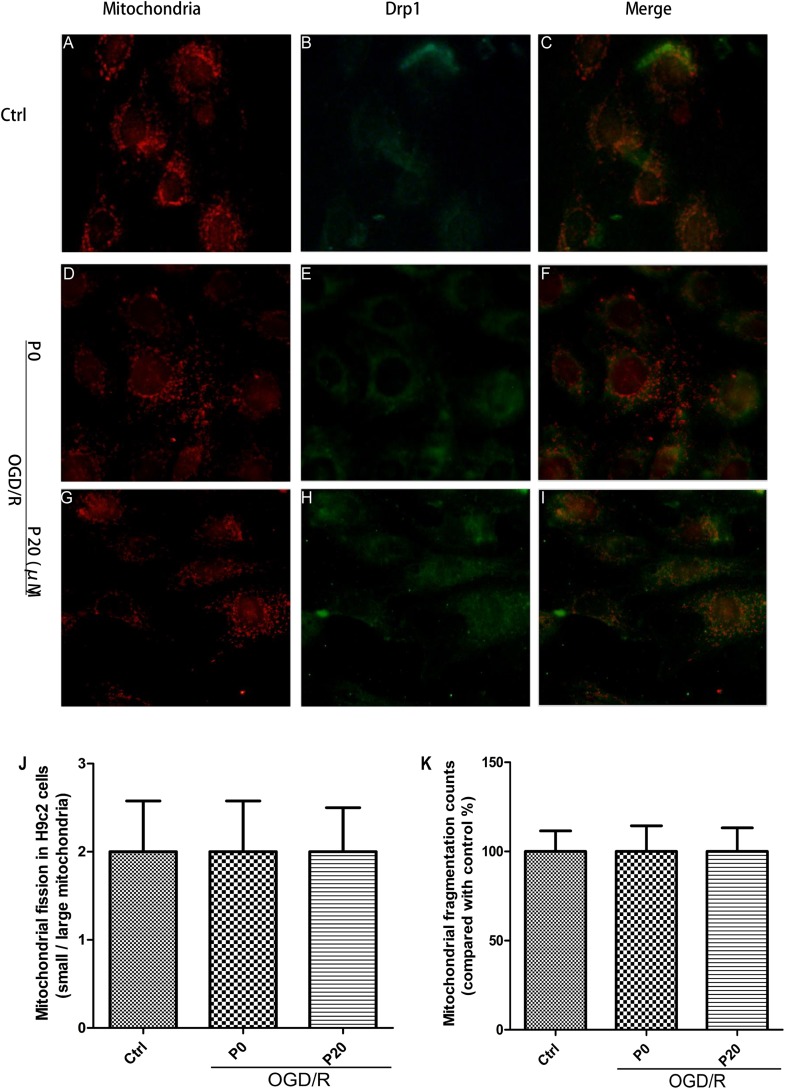
Mitochondrial morphology and quantity kept stable and propofol inhibited Drp1 colocalization with mitochondria in H9c2 cells during OGD/R injury. **(A,D,G,J,K)** Mitochondrial (red) morphology and quantity of H9c2 cells kept stable among control group, OGD/R group without propofol and OGD/R group with 20 μM propofol. Scale bar: 25 μm. **(B,C)** There was a lower fluorescence signal for Drp1 (green) and most Drp1 was collocated with the mitochondria or surrounded them in control group. **(E,H)** The distribution of Drp1 in OGD/R group with 20 μM propofol was more diffuse than the distribution of it in OGD/R group without propofol. **(F)** Plenty of Drp1 was collocated with mitochondria or surrounded them in OGD/R group without propofol. **(I)** Using 20 μM propofol treatment, Drp1 (green) was significantly prevented from cytoplasm to mitochondria during OGD/R injury. The data are presented as the mean ± SD of at least three independent experiments.

From the experimental results, we observed that the mitochondrial morphology and quantity of H9c2 cells remained stable among the control group, the OGD/R without propofol group and the OGD/R with 20 μM propofol group (Figure 5A,D,G,J,K). The result was consistent with the levels of mitochondrial membrane remodeling dynamin family proteins. Previous research has shown that Drp1 in other normal cells distributed throughout the cytoplasm, with lower-levels of colocalization with the mitochondria (Wang et al., 2015). However, surprisingly we found that most Drp1 was collocated with the mitochondria, or surrounded them in the H9c2 cells, whether in the control or OGD/R groups (Figure 5C,F,I). But there was a lower fluorescence signal for Drp1 in the control group (Figure 5B), which was consistent with lower levels of Drp1. Using 20 μM propofol treatment, there was more Drp1 in the cytoplasm of the H9c2 cells during OGD/R injury, compared with the group that received no propofol treatment (Figure 5E,F,H,I). The results suggested that plenty of Drp1 colocalizing with mitochondria or by surrounding them, worked in mitochondrial fission and propofol could significantly prevent Drp1 from cytoplasm to mitochondria.

### Propofol Could Inhibit Drp1 Phosphorylation Through Decreasing ERK Activation During OGD/R

**FIGURE 6:**
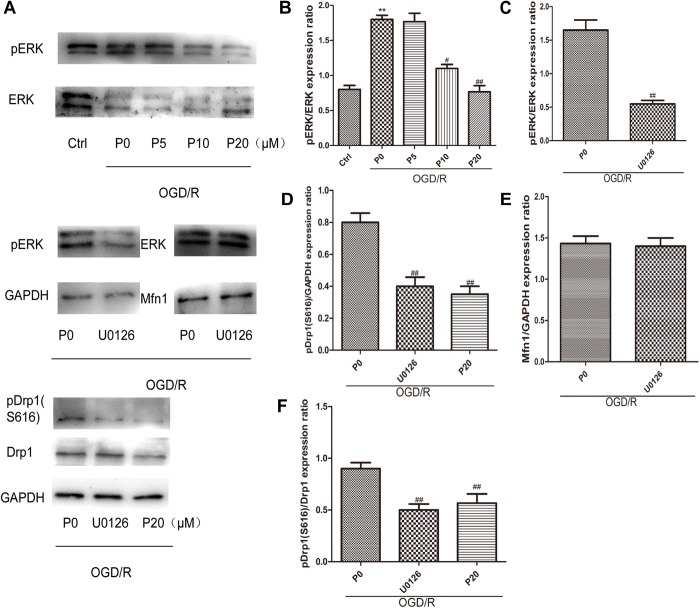
Propofol could inhibit Drp1 phosphorylation through decreasing ERK activation in H9c2 cells during OGD/R. **(A)** Representative bands of pERK, ERK, pDrp1, Drp1 and Mfn1 by western blotting. **(B)** With the increase of propofol concentration (10, 20 μM), the levels of pERK were significantly decreased during OGD/R injury. **(C)** U0126 can markedly inhibit ERK phosphorylation. **(D,F)** The levels of pDrp1 (S616) during OGD/R were significantly decreased by 20 μM propofol and U0126. **(E)** There was no significant difference in the levels of Mfn1 between OGD/R group without propofol and OGD/R group with U0126. The data are presented as the mean ± SD of at least three independent experiments. ^∗∗^*P* < 0.01 versus control, ^#^*P* < 0.05, ^##^*P* < 0.01 versus OGD/R group without propofol.

As previously reported, ERK is phosphorylated when oxidative stress occurs, relating to apoptosis, but its mechanism remains unclear (Kodiha et al., 2009; Rasola et al., 2010). The experimental results demonstrated that during OGD/R, the levels of pERK were significantly decreased by propofol in a dose-dependent manner at the range of 10∼20 μM (Figure 6A,B). Furthermore, U0126 can markedly inhibit ERK phosphorylation (Figure 6A,C). ERK1/2 have been reported to phosphorylate Drp1 at serine 616, resulting in increased mitochondrial fission (Qi et al., 2011; Yu et al., 2011; Strack et al., 2013). The levels of pDrp1 (S616) during OGD/R were significantly decreased by 20 μM propofol and U0126 (Figure 6A,D,F). Mfn1 was also found to be phosphorylated by ERK, which favored Bak oligomerization contributing to apoptosis (Pyakurel et al., 2015). U0126 could not influence the higher levels of Mfn1 in H9c2 cells induced by OGD/R (Figure 6A,E). These results demonstrated that propofol could inhibit Drp1 phosphorylation in H9c2 cells by decreasing ERK activation during OGD/R.

### Propofol Ameliorated H9c2 Cells Apoptosis During OGD/R via Inhibiting Mitochondrial Cytochrome c Release, Caspase-9, Caspase-6, Caspase-7, and Caspase-3 Activation

**FIGURE 7:**
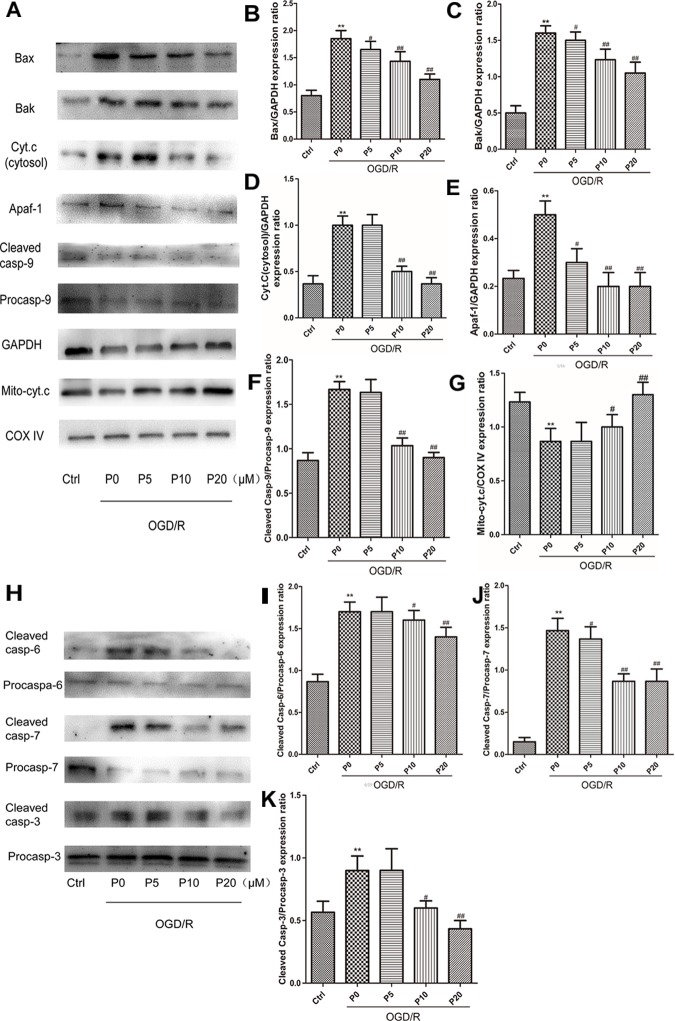
Propofol ameliorated H9c2 cells apoptosis during OGD/R via inhibiting intrinsic apoptosis pathway. **(A,H)** Representative bands of Bax, Bak, cytosol cytochrome c, Apaf-1, caspase-9, mitochondrial cytochrome c, caspase-6, caspase-7, and caspase-3 by western blotting. **(B,C,E,J)** With the increase of propofol concentration (5, 10, 20 μM), the levels of Bax, Bak, Apaf-1, and Cleaved caspase-7 were gradually reduced during OGD/R injury. **(D,F,I,K)** While the levels of cytosol cytochrome c, Cleaved caspase-9, Cleaved caspase-6 and Cleaved caspase-3 were markedly inhibited by 10 and 20 μM propofol during OGD/R injury. **(G)** And with the increase of propofol concentration (10, 20 μM), the level of mitochondrial cytochrome c protein was obviously elevated during OGD/R injury. The data are presented as the mean ± SD of at least three independent experiments. ^∗∗^*P* < 0.01 versus control, ^#^*P* < 0.05, ^##^*P* < 0.01 versus OGD/R group without propofol.

As is the case for most cells, the mitochondrial pathway (intrinsic pathway) is one of upstream apoptotic signaling. It involves the sequential release of cytochrome c, the recruitment of Apaf-1 (apoptotic protease-activating factor-1), and the activation of caspase-9 and downstream executioner caspases. The Bcl-2 family proapoptotic members Bax and Bak can mediate MOMP, releasing cytochrome c, along with other proapoptotic factors, into the cytosol (Frank et al., 2001). In the control group the levels of Bax, Bak, cytosol cytochrome c, Apaf-1, Cleaved caspase-9, Cleaved caspase-6, Cleaved caspase-7 and Cleaved caspase-3 were lower. Conversely, the levels of Bax, Bak, cytosol cytochrome c, Apaf-1, Cleaved caspase-9, Cleaved caspase-6, Cleaved caspase-7 and Cleaved caspase-3, were higher in the OGD/R without propofol group. In addition, with the increase of the propofol concentration (10, 20 μM), the levels of Bax, Bak, cytosol cytochrome c, Apaf-1, Cleaved caspase-9, Cleaved caspase-6, Cleaved caspase-7 and Cleaved caspase-3, were markedly reduced during OGD/R injury (Figure 7A–F,H–K). In the OGD/R without propofol group, the level of mitochondrial cytochrome c protein was significantly reduced compared with the control group. But with the increase of the propofol concentration (10, 20 μM), the level of mitochondrial cytochrome c protein was obviously elevated during OGD/R injury (Figure 7A,G). The experimental results demonstrated that propofol could ameliorate H9c2 cells apoptosis during OGD/R, via inhibiting the intrinsic pathway.

## Discussion

In the present study, we found that propofol could inhibit high levels of mitochondrial fission and fusion of the H9c2 cells, by regulating membrane remodeling dynamin family proteins during OGD/R injury and then ameliorate H9c2 cells apoptosis by the mitochondrial pathway. The concentration of propofol was consistent with those of previous investigations (Wang et al., 2009; Zhao et al., 2015). In our study, we observed that propofol at a concentration of 5 to 20 μM, significantly reduced cell death in a dose-dependent way and attenuated the level of ROS during OGD/R injury, which indicated that propofol had a strong protective effect against oxidative stress induced by I/R injury in cardiac myocytes. But when we treated H9c2 cells with propofol directly in a normal cell culture for 24 h, cell proliferation was obviously inhibited. And Z-VAD-FMK could relieve the decrease of cell viability induced by 20 μM propofol. This suggested that the protective effect of propofol was indeed due to the reduction of OGD/R damage, rather than the higher proliferation of the H9c2 cells during recovery. At the same time, it also demonstrated that high concentrations of propofol could cause damage to the H9c2 cells by activating caspases. As the concentration of propofol went up, propofol alleviated cell apoptosis induced by OGD/R in a dose-dependent manner, at the range of 5∼20 μM. Therefore, we further explored the mechanism of propofol inhibiting H9c2 cells apoptosis induced by OGD/R injury.

Previous studies showed that altering mitochondrial morphology affected cell apoptosis. Studies indicated that mitochondria underwent fission when HL-1 cardiac cells were subjected to simulated ischemia, generating fragmented mitochondria (Brady et al., 2006). We also observed that mitochondrial fission was obvious in H9c2 cells during OGD injury and propofol could significantly inhibited it. Mitochondrial ROS, which is generated during acute I/R injury is known to induce mitochondrial fission (Plotnikov et al., 2008). In our study, the level of ROS was higher in H9c2 cells during OGD/R injury. In the OGD/R without propofol group, the levels of Drp1, Fis1, Mfn1 and Mfn2 protein were significantly elevated compared with the control group. With the increase of propofol concentration (5, 10, 20 μM), the high levels of Drp1, Fis1, Mfn1 and Mfn2 protein during OGD/R injury were significantly decreased. The experimental results might demonstrate that mitochondrial fission and mitochondrial fusion were significantly elevated by OGD/R injury and were alleviated by propofol. In mitochondrial staining we observed that mitochondrial morphology and the quantity of H9c2 cells, remained stable in the control, OGD/R without propofol and OGD/R groups with 20 μM propofol. The result was consistent with the levels of mitochondrial membrane remodeling dynamin family proteins. Though mitochondrial fission was obvious in H9c2 cells during OGD injury, mitochondrial morphology and the quantity of H9c2 cells remained stable during OGD/R injury. This difference may be due to a high level of mitochondrial fusion during reperfusion.

It has been reported that propofol increases both the activities and protein expressions of superoxide dismutase and catalase to reduce intracellular ROS (Zhao et al., 2015). Additionally, it has been shown that mitochondrial fission induced by Drp1 could reduce inner membrane proton leak/uncoupling in other cell types, contributing to ROS production from mitochondria (Galloway et al., 2012a; Wang et al., 2015). Thus, propofol may reduce ROS production in H9c2 cells by decreasing the levels of Drp1 during OGD/R injury. ERK1/2 can be phosphorylated when oxidative stress occurs, which further phosphorylates Drp1 at serine 616, resulting in increased mitochondrial fission (Kodiha et al., 2009; Yu et al., 2011). Our results demonstrated that in immunofluorescence, plenty of Drp1 colocalizing with mitochondria or by surrounding them, worked in mitochondrial fission. Moreover, propofol could inhibit Drp1 phosphorylation at serine 616 in H9c2 cells by decreasing ERK activation during OGD/R, thus resulting in inhibiting mitochondrial fission.

A previous study also showed that genetic inhibition of Drp1 in HL-1 cardiac cells, not only inhibited the mitochondrial fission induced by acute I/R injury but also reduced cell death in this setting (Ong et al., 2010). Drp1 has been demonstrated to co-localize with Bax at the outer mitochondrial membrane in response to apoptotic stimuli (Karbowski et al., 2002). Bax assembles with Bak and Drp1 in the form of a ring, to delineate the area required for MOMP (Grosse et al., 2016). Interestingly, we found that propofol could inhibit the high expression of Drp1, Fis1, Bax and Bak proteins induced by OGD/R injury. Fis1 has been demonstrated to promote the migration of Drp1 to mitochondria in the cytoplasm (Stojanovski et al., 2004). We observed that in immunofluorescence, propofol can significantly prevent Drp1 from cytoplasm to mitochondria during OGD/R injury. Therefore, propofol could reduce Drp1 colocalization with mitochondria by lowering levels of Drp1 and Fis1. Propofol may inhibit outer membrane permeabilization by reducing the high levels of Drp1, Bax and Bak during OGD/R injury.

When Mfn1 is phosphorylated by ERK, mitochondria fragment and Bak oligomerization is favored, therefore cytochrome c release and cell death (Pyakurel et al., 2015). Our results demonstrated that propofol could significantly decrease ERK activation in H9c2 cells during OGD/R injury. U0126 did not influence the higher levels of non-phosphorylated Mfn1 in H9c2 cells induced by OGD/R. We speculate that propofol could still inhibit Mfn1 phosphorylation in H9c2 cells, by decreasing ERK activation during OGD/R injury. In the heart, acceleration of Mfn1 degradation by membrane-associated RING-CH (MARCH5) under stress, remains an important quality control system that inhibits cell death (Park et al., 2014). Oxidative stress with H_2_O_2_ leads to concurrent increases in Mfn2 expression and apoptosis in cultured neonatal rat cardiomyocytes, and Mfn2 silencing inhibits oxidative stress-induced apoptosis in H9c2 cells. Mfn2 also suppresses phosphatidylinositol 3 kinase-protein kinase B (PI3K-Akt) cell survival signaling pathways (Shen et al., 2007). Mfn2 has been demonstrated to co-localize with both Bax and Bak in the outer mitochondrial membrane (Neuspiel et al., 2005). Our data indicated that propofol could inhibit the high expression of the Mfn1 and Mfn2 protein induced by OGD/R injury. OPA1 mediates mitochondrial inner membrane fusion and regulates mitochondrial cristae morphology (Olichon et al., 2003; Cipolat et al., 2004). Anti-apoptotic effects of Opa1 have been attributed to its role in regulating mitochondrial cristae morphology and cytochrome c distribution (Korwitz et al., 2016). However, in our study, propofol did not influence the levels of Opa1 during OGD/R injury and there were no significant changes of Opa1 in OGD/R injury. This could be because the H9c2 cells were less damaged, which did not damage the mitochondrial inner membrane, but affected the mitochondrial outer membrane.

**FIGURE 8:**
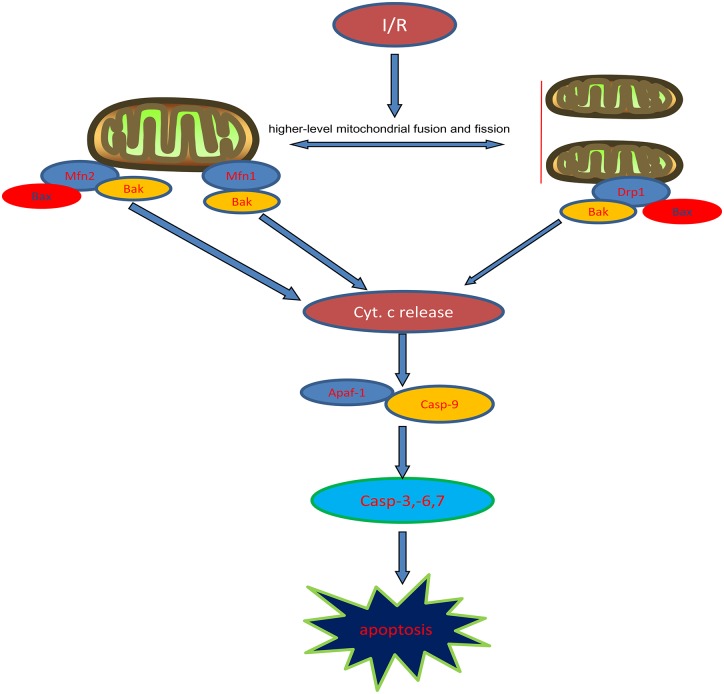
Diagram of mitochondrial dynamics regulating intrinsic apoptosis pathway.

To further test that propofol inhibits higher outer mitochondrial membrane permeabilization induced by OGD/R injury, we measured the levels of cytochrome c in the cytoplasm or mitochondria. Our results indicated that cytochrome c was released into the cytoplasm and the process was inhibited by propofol during OGD/R injury. Cytochrome c, Apaf-1 and procaspase-9 in the cytoplasm combine to form the apoptosome, which leads to procaspase-9 activation (Srinivasula et al., 1998; Zhang et al., 2002). Active caspase-9 then activates the executioner caspase-3, -6, and -7 to complete apoptosis. We found that the expression levels of Apaf-1 and the activities of caspase-9, -3, -6, and -7 were higher in H9c2 cells during OGD/R injury, which could be reversed by propofol. This demonstrated that propofol could reduce H9c2 cells apoptosis during OGD/R injury by inhibiting the mitochondria pathway, which was due to reducing high levels of mitochondrial fusion and fission induced by OGD/R injury (Figure 8).

However, all our experimental results were limited to the level of cells *in vitro*, and the study on the regulation of propofol on mitochondrial morphology was limited to the regulation of the protein level. Further animal experiments need to be conducted to verify our experimental results, and the effect of propofol on mitochondrial fusion and fission may be studied from other regulatory levels. In short, our study may reveal a new cardioprotective mechanism of propofol during acute ischemia/reperfusion injury.

## Author Contributions

LZ wrote the manuscript and conceived and conducted the entire study. ZW, JH, and YW wrote the manuscript and amended the references. JZ, DZha, and DZho edited the whole article and offered technical support. HZ, WZ, and MY offered technical assistance and reviewed the text. SZ and JP performed the experiments and analyzed the data. All authors have read and approved the manuscript.

## Conflict of Interest Statement

The authors declare that the research was conducted in the absence of any commercial or financial relationships that could be construed as a potential conflict of interest.
